# A systematic review on how to diagnose deltoid ligament injuries—are we missing a uniform standard?

**DOI:** 10.1186/s12891-024-07869-1

**Published:** 2024-10-03

**Authors:** Judith Schrempf, Sebastian Baumbach, Nasef Mohamed N. Abdelatif, Hans Polzer, Wolfgang Böcker

**Affiliations:** 1 Musculoskeletal University Centre Munich (MUM), University Hospital, Ludwig-Maximilians-University Munich (LMU), Munich, Germany; 2 Orthopedic Department OrthoClinic, Cairo, Egypt

**Keywords:** Deltoid ligament injury, Deltoid ligament imaging, Deltoid ligament rupture, Deltoid ligament diagnosic, Medial ankle instability, Collateral ligaments, Systematic review, Ankle joint

## Abstract

**Background:**

Up to now, there is no convincing evidence, that surgical treatment of deltoid ligament injuries, especially in the setting of ankle fractures, does result in improved outcome. One reason could be a missing diagnostic standard. The aim of the current systematic review was to analyze the applied diagnostic strategies for acute deltoid ligament injuries in outcome studies.

**Methods:**

MEDLINE, Scopus, Central, and EMBASE were searched through February 2022 for any original studies addressing diagnostics of acute deltoid injuries. The study was conducted per the PRISMA guidelines. The inclusion criteria were formed according to the PICOS criteria. The data assessed were study type, level of evidence, included fractures, time point and method of diagnosing deltoid ligament layers, differentiation between layers and syndesmotic injuries.

**Results:**

31 studies were included in the final analysis. Most studies (*n* = 28) based their decision to treat the deltoid ligament injury on radiologic findings only, with stressed radiographs (*n* = 18) being the most common. The radiographs were applied at one or more time points (preoperative, before ORIF, after ORIF, after ORIF and syndesmotic repair). The most frequently assessed parameter was the Medial Clear Space (MCS, *n* = 27) with cut-off-values considered pathological ranging between MCS > 1 mm and MCS > 6 mm.

**Conclusion:**

Comparing the 31 studies shows that a standardized method to diagnose deltoid ligament injuries is missing. Further research is needed to establish evidence-based guidelines on how to diagnose acute deltoid ligament injuries.

**Trial registration:**

Prospero ID: CRD42022307112.

Clinical trial number: not applicable.

**Supplementary Information:**

The online version contains supplementary material available at 10.1186/s12891-024-07869-1.

## Introduction

The ankle joint is a complex joint, stabilized by its bony geometry as well as several ligamentous structures. One of the indispensable stabilizers is the deltoid ligament bundles. It centers the talus under the tibia, prevents lateral talar translation, and provides rotational stability [7,20,41,44].

The deltoid ligament bundle comprises a superficial (SDL) and deep (DDL) layer. The SDL comprises four, the DDL of two components, originating from the medial malleolus, spanning to the talus, calcaneus, spring ligament, and navicular bone [28,44]. Acute deltoid ligament injuries most often present in combination with ankle fractures [14,19,32]. To date there is no clear consensus that surgical treatment of an acute deltoid ligament injury results in superior patient rated outcome [14,22,29,52].

Interestingly, there appears to be a considerable knowledge gap on how to diagnose and rate the extent of an injury to the deltoid ligament complex. The applied diagnostic tools range from non-weightbearing radiographs to MRI, with again varying cut-off values [21,49,53,60]. Moreover, almost no study differentiates between the different layers or bundles of the deltoid ligament injured. Accordingly, efficacy of different treatment strategies is not comparable if injury patterns are not defined accurately.

The aim of the current study therefore was to assess the status quo of diagnostics applied in published outcome studies on deltoid ligament injuries.

## Materials and method

The systematic review followed the Preferred Reporting Items for Systematic Reviews (PRISMA) guidelines [40] and was published a priory at Prospero (Prospero ID: CRD42022307112). The initial protocol was adapted due to an emerging number of other systematic reviews focusing on the patient rated outcome. Therefore, the authors decided to focus their systematic review on the applied diagnostics. The Prospero protocol was changed accordingly.

### Search strategy

The research question was built according to the PICOS criteria (Table 1).

**Table 1:** PICOS criteria defining the inclusion and exclusion criteria

**P**opulation	Skeletal mature patients with an acute injury to the deltoid ligament complex, either isolated or in combination with an ankle fracture and/or syndesmotic injury
**I**ntervention	Conscious decision to either treat or not treat an acute injury to the deltoid ligament. Studies must report on their approach how to diagnose deltoid ligament injury
**C**omparison	not applicable
**O**utcomes	Any objective outcome parameters such as radiographic measurements, clinical data or patient rated outcome scores
**S**tudy	Eligible were any English written cadaver/biomechanical or clinical studies, regardless of the study design, with at least 10 patients included

The search query was built upon the concepts “Ligament” AND “Deltoid” AND “Injury / Rupture / Imaging / Diagnosis”. The detailed search strategies are presented in Supplement 1. MEDLINE (PubMed), Scopus, Central and EMBASE were searched for original studies published from inception to February 2022. A grey literature search for conference proceedings in both Scopus and EMBASE was performed. Furthermore, the reference lists of other systematic reviews as well as those of papers included in this systematic review were hand-searched for additional eligible studies.

### Study selection and data extraction

The search results of each database were exported to EndnoteTM (Vs. 20.1, Fa. Clarivate). Based on the standard EndnoteTM algorithm, duplicates were removed. The final dataset was imported into CovidenceTM (Melbourne, Australia) which again removed duplicates. The further study selection process was conducted by two independent reviewers (JS, ANMN) within Covidence™. Conflicts were resolved by a third reviewer (SFB).

A data extraction sheet was built in Excel (JS, ANMN) and was filled by two independent reviewers (JS, ANMN). The data assessed were study type (e.g., case series, retrospective cohort, randomized clinical trial), the level of evidence, and methods of diagnosis, (included fractures, syndesmotic injuries, differentiation of layers). The two data sheets were finally merged, and the disagreement was resolved by discussion (JS, ANMN, SFB).

### Risk of bias assessment

The studies’ level of evidence was assessed according to the criteria published by Wright et al. [56]. The risk of bias was assessed by The Methodological Index for Non-randomized Studies (MINORS), which has also been validated for randomized controlled trials [47].

### Data analysis

A descriptive analysis was performed for the different diagnostics applied, as well as for the individual cut-off values used. These were listed separately per the time they were assessed, i.e. preoperative, before open reduction and internal fixation (ORIF), after ORIF, and after ORIF and syndesmotic stabilization.

## Results

**Fig. 1:**
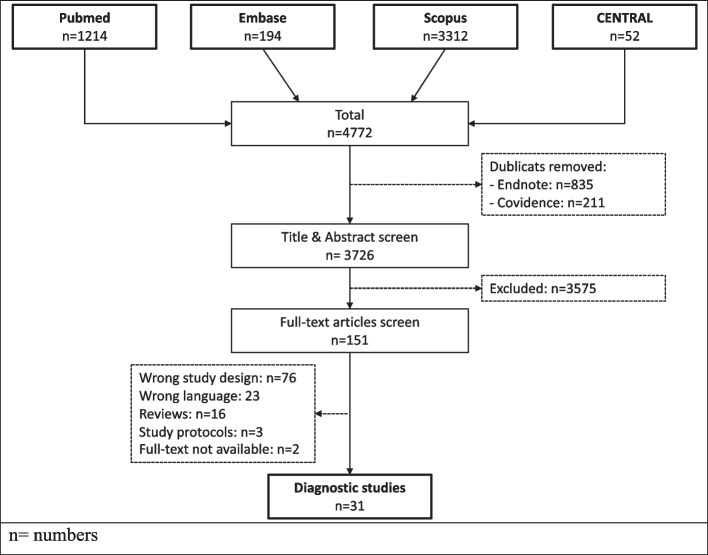
Study selection flow chart according to the PRISMA guidelines

The study selection process is depicted per the PRISMA recommendations in Fig. 1. Out of 3726 studies, 31 studies were eligible. Biomechanical studies were excluded due to a missing diagnostic approach in these studies. All 31 studies included fracture cases. No study on isolated, acute deltoid ligament injuries meeting the herein defined criteria could be identified. Twenty-six studies conducted some sort of deltoid ligament repair [2–4,8,9,12,16,18,21,26,27,31,33–35,37,42,46,48–50,53,55,57,58,60]. The treatment approaches included suture anchor(s), [4,8,12,18,21,26,27,31,33,35,37,38,42,45,46,48,50,53,55,57,58,60] and/or direct suture [3,6,8,12,16,27,49,55,57,58,60]. One study used a anterior tibialis tendon graft for repair [23]. Five studies consciously only addressed the bony injuries and did not treat the deltoid ligament injury [5,24,30,51,59]. Reasons for not addressing the unstable deltoid ligament were a purely diagnostic approach [5]. Three studies assessed the patient rated outcome following untreated deltoid ligament injury, [25,30,59] and Teijwani et al. compared the outcome of functional bimalleolar injuries to actual bimalleolar fracutres [51].

Per the MINORS criteria, the non-randomized, non-comparative studies reached 8/16 points (*n* = 10), comparative studies 13/24 points (*n* = 16), and RCTs 15/24 points (*n* = 5) on average, a detailed overview is depicted in Supplement 2. A detailed overview of all 31 studies included is provided in Supplement 3.

**Fig. 2:**
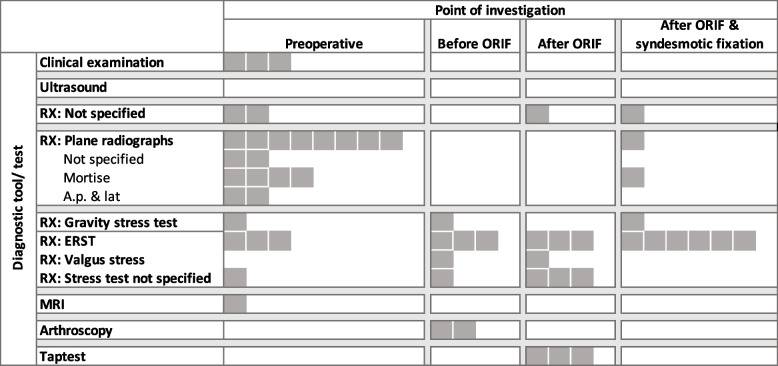
Frequency of the applied diagnostics according to different time points

An overview of the individual diagnostic tools per the time points they were applied is outlined in Fig. 2. Each grey square resembles a study in which the diagnostic tool was applied as part of their assessment routine to set the indication to address the deltoid ligament injury. If a single study applied several diagnostic tools, it is resembled by several grey squares. The number of squares therefore does not resemble the number of studies, but the number of diagnostic tools applied. Most studies (90%; *n* = 28) based their indication to operate on the deltoid ligament on radiologic diagnostics [3–5,8,9,12,16,18,21,25,27,30,31,33–35,37,42,46,48–51,53,55,57,58,60], the remaining 10% (*n* = 3) on a combination of radiologic- and clinical examination findings [2,26,59]. The clinical findings used were swelling, pain and tenderness in two studies, [31,59] hematoma/bruising, and a positive dimple sign in one study [26]. Another eight studies reported on clinical findings but did not incorporate them into their diagnostic algorithm [3–5,9,37,55,57,58]. Twenty-seven studies based their radiologic diagnostics solely on radiographs: 15 studies facilitated stressed radiographs [2,5,18,27,31,33–35,42,46,48,50,51,53,55], seven studies unstressed radiographs [3,12,25,37,49,59,60], one study stressed and unstressed radiographs [58], and four studies did not specify which type of radiographs were used [8,16,30,57]. The remaining four studies used stressed radiographs and MRI [21] (*n* = 1), stressed radiographs and arthroscopy [26] (*n* = 1), arthroscopy [4] (*n* = 1) or sonography [9] (*n* = 1).The most applied stressed radiography was the external rotation stress test [2,26,31,34,42,46,48,50,51,53,55,58] (*n* = 12). Other facilitated tests, either solely or additionally, were the gravity stress test, [5,31,55] valgus stress test [33,50], and the tap test [5,57]. Four studies did not further specify which stress test was used [18,21,27,35].

81% of studies set the indication at one time point (preoperative [2,8,9,12,21,25,31,37,49,51,59,60] (*n* = 12), intraoperative before ORIF [4,17,18,50] (*n* = 4), after ORIF [16,27,33] (*n* = 3), after ORIF and syndesmotic repair [3,30,34,42,46,55] (*n* = 6)), the remaining six studies reevaluated the indication at different time points [5,26,35,48,57,58]. Overall, 18 out of the 31 studies included, differentiated between a syndesmotic and deltoid ligament injury [3,4,8,12,18,26,27,33,35,37,42,46,48,50,51,55,57,60].

**Fig. 3:**
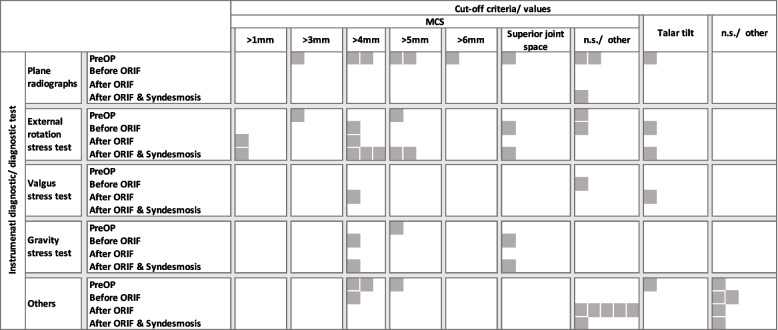
Cut-off parameter according to the applied diagnostics and time points

Next, the individual radiographic tools were analyzed per the chosen cut-off parameter (Fig. 3). Similar to Fig. 2, each grey square resembles one diagnostic. Therefore, a single study can be resembled by multiple squares. The most commonly assessed parameter was the MCS (*n* = 27), either solely [2,3,5,8,12,16,18,21,25,31,35,37,42,46,48–51,53,55,59,60] (*n* = 22), or in combination with other parameters [26,33,34,57,58] (*n* = 5). The remaining studies either used arthroscopy,^4^ ultrasonography [8], or did not further specify the parameter used [27,30].

The cut-off criteria for the MCS varied between MCS > 1mm [58] and MCS > 6 mm, [2,5,8,12,18,21,25,31,34,37,42,46,48,52,53,55,57,58,60] and/ or was compared to the superior joint space [5,49,50,53,55] / lateral clear space [49]. Four studies used the talar tilt, [26,33,34,57] out of which only one study stated an actual cut-off value (more than two degrees) [33]. Seven studies did not specify a cut-off value [3,9,16,26,35,51,59].

Overall, two studies (9%) differentiated between the DDL and SDL preoperatively, either by MRI [33] or sonography [9], 20 studies (65%) did not differentiate between the SDL and DDL for diagnostic purposes [3,5,12,16,18,21,25,30,31,34,37,42,46,48,49,51,53,57–59] and eight studies (26%) differentiated SDL and DDL intraoperatively by direct visualization [2,4,8,27,35,50,55,60]. The authors of one study [26] were contacted via email and stated, that they diagnosed an SDL injury pre-surgically clinically and a DDL injury intraoperatively via arthroscopy.

## Discussion

Based on a systematic literature review, the authored identified 31 studies reporting on diagnostic strategies to identify deltoid ligament injuries. These studies revealed a significant inconsistency, with respect to the tests applied, the chosen cut-off criteria and the time points of assessment. Consequently, we are still missing a uniform standard to diagnose deltoid ligament instability.

To date, the available literature is inconclusive on whether a deltoid ligament repair in ankle fracture cases result in a superior outcome, or not. Some comparative studies have advocated not repairing the associated deltoid injury if the fibula and syndesmosis were adequately reduced and the anatomical position of the talus was restored [3,36,49,50]. Other investigators have recommended to explore and repair the deltoid [31,55,57,60]. One reason for these inconclusive findings could be varying diagnostic strategies. The current study aimed to investigate this heterogeneity in diagnostic strategies in studies reporting on the outcome of deltoid ligament injuries.

The diagnostic strategies applied in the studies analyzed revealed an astonishing heterogeneity. Overall, no standard diagnostic approach or cut-off values could be identified.

Compared to conventional radiographs, direct visualization via arthroscopy is considered the most reliable diagnostic approach, especially for the DDL [1,10,11]. Still, arthroscopy is an invasive procedure, which could be hard to argue, as final data on the efficiency of deltoid ligament repair, at least in fracture cases, are still missing [29]. MRI often is believed the non-invasive gold standard to assess ligamentous injuries. Crim et al. conducted a comparative study and reported a sensitivity/specificity for SDL injuries of 83%/94% and for the DDL 69%/98% for MRI compared to intraoperative findings [13]. Other studies were able to show, that the sensitivity/specificity of MRI is further impaired in fracture cases [15,39,54]. Finally, although MRI is good at indicating an injury to a ligament, it has limitations in distinguishing between an injury and a complete rupture [32]. Beside to those imaging limitations, MRI is expensive and not widely available compared to radiography or ultrasound. Therefore, there is a need for a reliable, easily available diagnostic tool to identify deltoid ligament injuries, best separate for SDL and DDL injuries.

Only two out of the 31 included studies did use arthroscopy [4,26] and only one study used MRI [21] to diagnose a deltoid ligament rupture. None of the studies included based their indication to address the medial side on the clinical examination alone. This is not surprising, as previous studies were able to show its limited value [17]. The most used apparative diagnostic modality were radiographs, either unstressed [3,12,24,37,49,59,60] (*n* = 7) or stressed [2,5,18,27,31,33–35,42,46,48,50,51,53,55,58] (*n* = 16). The ERST was the most applied stressed radiograph [2,26,31,34,42,46,48,50,51,53,55,58] (*n* = 12).

For both, unstressed and stressed radiographs, the most assessed parameter was the MCS. Still, cut-off values for the MCS ranged between MCS > 1 mm and MCS > 6 mm, with a MCS widening of 4 mm being the most used value. However, DeAngelis et al. were able to show a false-positive deltoid rupture rate of 54% when using 4 mm MCS as a cut-off value, when compared to arthroscopy [17]. The most applied stress radiography was the ERST under fluoroscopy [2,26,31,34,42,46,48,50,51,53,55,58] (*n* = 12). Interestingly, the 4 mm cut-off value was applied for different time points throughout the surgery, i.e. before ORIF, after ORIF, and after ORIF and stabilization of the syndesmosis. One would assume, that with an increase in mortise stability during the surgical treatment, the MCS cut-off value should change. Cheung et al. argued to assess deltoid ligament stability after ORIF of the bony injuries and syndesmotic stabilization, as the MCS assessed during the ERST is also influenced by an syndesmotic instability [10].

A promising, but still considerably underrated diagnostic alternative is ultrasound. Similar to radiography and MRI, it is non-invasive, but it can be performed dynamically. A dynamic examination technique, as stress radiographs, allows to visualize a possible dynamic instability, which often cannot be delineated in static examination techniques, such as unstressed radiographs or MRI. Rosa et al. have reported a sensitivity/specificity for ultrasonographic examination in fracture cases as high as 100%/90% [43]. Still, only one out of thirty examined studies study identified in the current systematic review did use sonography [9].

The huge diagnostic gap for deltoid ligament injuries becomes even more evident when one does consider, that it would be desirable to differentiate between SDL, DDL or combined injuries. Per the current systematic review, only less than a third of the studies (nine studies) differentiated between the SDL and DDL in their diagnostics, [2,4,8,26,27,35,50,55,60] with again great heterogeneity.

De Krom et al. [15] conducted a systematic review and meta-analysis including studies that specifically reported on the sensitivity/specificity and positive/negative predictive value of any diagnostic tool to diagnose deltoid ligament rupture. They concluded that “Ultrasonography and gravity stress radiography seem the most accurate diagnostic tools”. But when looking at the individual reference test of the included studies (Manual ERST: *n* = 5, Gravity stress test: *n* = 2; MRI: *n* = 2; Radiography/Arthrography/intraoperative visualization: *n* = 1 each) it becomes evident, that these were not only heterogenic, but all miss a valid reference test, i.e. arthroscopy. Therefore, the authors believe that the data available neither allow to conduct a meta-analysis nor do they allow to draw a conclusion on the “most accurate diagnostic tool”.

Considering the data currently available in the literature, we are missing a non-invasive standard to diagnose SLD and/or DDL injuries. The currently applied diagnostics in outcome studies are heterogeneous to the extent, that these studies are close to incomparable.

The major limitation of the current study is the above outlined heterogeneity of the studies available. Further limitations are the restriction to English language, and the exclusion of studies with less than 10 patients. Still, the authors followed the strict criteria for systematic reviews.

## Conclusion

The diagnosis of deltoid ligament injuries and instability is still missing a consensus. Literature is lacking exact definitions for deltoid instability, diagnostic measures, cut-off values and even the timing of the measurements for this injury. Evidently, high-level randomized trials in addition to adequately performed biomechanical studies are in abundant demand.

## Supplementary Information

Supplementary Material 1.

Supplementary Material 2.

Supplementary Material 3.

## Data Availability

All data analysed during this study is provided within the manuscript and supplementary information files.

## References

[1] Arthur D, et al. Correlating arthroscopic and radiographic findings of deep deltoid ligament injuries in rotational ankle fractures. Foot Ankle Int. 2021;42(3):251–6. 33106030 10.1177/1071100720962796

[2] Asadi K, Mardani-Kivi M. Orod G, Deltoid ligament reconstruction in lateral malleolus fractures with deltoid rupture. Trauma Monthly. 2021;26(3):135–40.

[3] Baird RA, Jackson ST. Fractures of the distal part of the fibula with associated disruption of the deltoid ligament Treatment without repair of the deltoid ligament. J Bone Joint Surg Am. 1987;69(9):1346–52. 3440794

[4] Barbachan Mansur NS, et al. Deltoid ligament arthroscopic repair in ankle fractures: Case series. Injury. 2021;52(10):3156–60. 34247766 10.1016/j.injury.2021.06.020

[5] Bi C, et al. Diagnostic value of intraoperative tap test for acute deltoid ligament injury. Eur J Trauma Emerg Surg. 2021;47(4):921–8. 31624856 10.1007/s00068-019-01243-w

[6] Butler BA, et al. Deltoid ligament repair reduces and stabilizes the talus in unstable ankle fractures. J Orthop. 2020;17:87–90. 31879481 10.1016/j.jor.2019.06.005 PMC6919363

[7] Campbell KJ, et al. The ligament anatomy of the deltoid complex of the ankle: a qualitative and quantitative anatomical study. J Bone Joint Surg Am. 2014;96(8): e62. 24740670 10.2106/JBJS.M.00870

[8] Chen H, et al. The importance of the deep deltoid ligament repair in treating supination-external rotation stage IV ankle fracture: a comparative retrospective cohort study. Biomed Res Int. 2020;2020:2043015. 33313312 10.1155/2020/2043015 PMC7719498

[9] Chen PY, Wang TG, Wang CL. Ultrasonographic examination of the deltoid ligament in bimalleolar equivalent fractures. Foot Ankle Int. 2008;29(9):883–6. 18778665 10.3113/FAI.2008.0883

[10] Cheung Y, et al. MRI of isolated distal fibular fractures with widened medial clear space on stressed radiographs: which ligaments are interrupted? AJR Am J Roentgenol. 2009;192(1):W7-12. 19098171 10.2214/AJR.08.1092 PMC5585779

[11] Chiang CC, et al. Arthroscopic quantitative measurement of medial clear space for deltoid injury of the ankle: a cadaveric comparative study with stress radiography. Am J Sports Med. 2022;50(3):778–87. 35289224 10.1177/03635465211067806

[12] Choi S, et al. Does repair of deltoid ligament contribute to restoring a mortise in SER type IV ankle fracture with syndesmotic diastasis? Arch Orthop Trauma Surg. 2022;142(4):535–41. 33119800 10.1007/s00402-020-03645-7

[13] Crim J, Longenecker LG. MRI and surgical findings in deltoid ligament tears. AJR Am J Roentgenol. 2015;204(1):W63–9. 25539277 10.2214/AJR.13.11702

[14] Dabash S, et al. Adding deltoid ligament repair in ankle fracture treatment: Is it necessary? A systematic review. Foot Ankle Surg. 2019;25(6):714–20. 30482440 10.1016/j.fas.2018.11.001

[15] de Krom MA, et al. Diagnostic tools to evaluate ankle instability caused by a deltoid ligament rupture in patients with supination-external rotation ankle fractures: A systematic review and meta-analysis. Injury. 2022;53(2):724–31. 34602247 10.1016/j.injury.2021.09.034

[16] de Souza LJ, Gustilo RB, Meyer TJ. Results of operative treatment of displaced external rotation-abduction fractures of the ankle. J Bone Joint Surg Am. 1985;67(7):1066–74. 3928632

[17] DeAngelis NA, Eskander MS, French BG. Does medial tenderness predict deep deltoid ligament incompetence in supination-external rotation type ankle fractures? J Orthop Trauma. 2007;21(4):244–7. 17414551 10.1097/BOT.0b013e3180413835

[18] Diab HSM. Suture anchor repair for ruptured deltoid ligament in pronation ankle fractures. Curr Orthop Pract. 2017;28(5):459–64.

[19] Ebraheim NA, Elgafy H, Padanilam T. Syndesmotic disruption in low fibular fractures associated with deltoid ligament injury. Clin Orthop Relat Res. 2003;409:260–7. 10.1097/01.blo.0000052935.71325.30 12671510

[20] Golano P, et al. Anatomy of the ankle ligaments: a pictorial essay. Knee Surg Sports Traumatol Arthrosc. 2010;18(5):557–69. 20309522 10.1007/s00167-010-1100-x PMC2855022

[21] Gu G. Efficacy of deltoid ligament reconstruction on the curative effect, complication and long-term prognosis in ankle fracture-dislocation with deltoid ligament injury. Int J Clin Exp Med. 2017;10(9):13778–83.

[22] Guo W, et al. Comparison of deltoid ligament repair and non-repair in acute ankle fracture: A meta-analysis of comparative studies. PLoS ONE. 2021;16(11):e0258785. 34767584 10.1371/journal.pone.0258785 PMC8589189

[23] Haddad SL, et al. Deltoid ligament reconstruction: a novel technique with biomechanical analysis. Foot Ankle Int. 2010;31(7):639–51. 20663434 10.3113/FAI.2010.0639

[24] Harper MC. Deltoid ligament: an anatomical evaluation of function. Foot Ankle. 1987;8(1):19–22. 3623356 10.1177/107110078700800104

[25] Harper MC. The deltoid ligament An evaluation of need for surgical repair. Clin Orthop Relat Res. 1988;226:156–68. 3121227

[26] Hsu AR, Lareau CR, Anderson RB. Repair of acute superficial deltoid complex avulsion during ankle fracture fixation in national football league players. Foot Ankle Int. 2015;36(11):1272–8. 26160387 10.1177/1071100715593374

[27] Isabel Rosa JR. Pedro Xavier Fernandes, Raquel Teixeira, Hugo Ribeiro, José Guimarães Consciência, Comparison of deltoid ligament repair and syndesmotic fixation in malleolar fractures. Sci J Foot Ankle. 2019;13(3):205–11.

[28] Ismail EE Sr, et al. Defining the Components of the Deltoid Ligament (DL): A Cadaveric Study. Cureus. 2022;14(3):e23051. 35464563 10.7759/cureus.23051 PMC9001815

[29] James M, Dodd A. Management of deltoid ligament injuries in acute ankle fracture: a systematic review. Can J Surg. 2022;65(1):E9–15. 35017184 10.1503/cjs.020320 PMC8759295

[30] Johnson DP, Hill J. Fracture-dislocation of the ankle with rupture of the deltoid ligament. Injury. 1988;19(2):59–61. 3198264 10.1016/0020-1383(88)90071-x

[31] Jones CR, Nunley JA 2nd. Deltoid ligament repair versus syndesmotic fixation in bimalleolar equivalent ankle fractures. J Orthop Trauma. 2015;29(5):245–9. 25186845 10.1097/BOT.0000000000000220

[32] Lee S, et al. Deltoid Ligament Rupture in Ankle Fracture: Diagnosis and Management. J Am Acad Orthop Surg. 2019;27(14):e648–58. 30475279 10.5435/JAAOS-D-18-00198

[33] Lee TH, et al. The contribution of anterior deltoid ligament to ankle stability in isolated lateral malleolar fractures. Injury. 2016;47(7):1581–5. 27133289 10.1016/j.injury.2016.03.017

[34] Li B, et al. Transarticular external fixation versus deltoid ligament repair in treating SER IV ankle fractures: a comparative study. BMC Musculoskelet Disord. 2019;20(1):453. 31627717 10.1186/s12891-019-2840-5 PMC6800498

[35] Li T, et al. Clinical Study of Ankle Fracture Combined With Deltoid Ligament Injury: Repair or Not? A Retrospective. Comparative Study J Foot Ankle Surg. 2020;59(4):648–52. 32600557 10.1053/j.jfas.2018.07.005

[36] Maynou C, et al. Is surgical treatment of deltoid ligament rupture necessary in ankle fractures? Rev Chir Orthop Reparatrice Appar Mot. 1997;83(7):652–7. 9515134

[37] Mirza Zafer Dağtaş, Ö.K.Ü., Deltoid Ligament Repair in Addition to Syndesmotic Fixation in Distal Fibula Fractures is Associated with Better Clinical Results in Mid- and- Long-term Follow-up: A Comparative Study. Bagcilar Med Bull 2021;6(4):431–437.

[38] Mococain P, et al. Biomechanical effect on joint stability of including deltoid ligament repair in an ankle fracture soft tissue injury model with deltoid and syndesmotic disruption. Foot Ankle Int. 2020;41(9):1158–64. 32545997 10.1177/1071100720929007

[39] Nortunen S, et al. Stability assessment of the ankle mortise in supination-external rotation-type ankle fractures: lack of additional diagnostic value of MRI. J Bone Joint Surg Am. 2014;96(22):1855–62. 25410502 10.2106/JBJS.M.01533

[40] Page MJ, et al. The PRISMA 2020 statement: an updated guideline for reporting systematic reviews. BMJ. 2021;372: n71. 33782057 10.1136/bmj.n71 PMC8005924

[41] Pankovich AM, Shivaram MS. Anatomical basis of variability in injuries of the medial malleolus and the deltoid ligament I Anatomical studies. Acta Orthop Scand. 1979;50(2):217–23. 107719 10.3109/17453677908989759

[42] Park YH, et al. Comparison of outcome of deltoid ligament repair according to location of suture anchors in rotational ankle fracture. Foot Ankle Int. 2021;42(1):62–8. 32951566 10.1177/1071100720952053

[43] Rosa I, et al. Ultrasonographic assessment of deltoid ligament integrity in ankle fractures. Foot Ankle Int. 2020;41(2):147–53. 31597464 10.1177/1071100719882679

[44] Savage-Elliott I, et al. The deltoid ligament: an in-depth review of anatomy, function, and treatment strategies. Knee Surg Sports Traumatol Arthrosc. 2013;21(6):1316–27. 22878436 10.1007/s00167-012-2159-3

[45] Schottel PC, et al. Anatomic ligament repair restores ankle and syndesmotic rotational stability as much as syndesmotic screw fixation. J Orthop Trauma. 2016;30(2):e36-40. 26313231 10.1097/BOT.0000000000000427

[46] Shen JJ, et al. Suture anchors for primary deltoid ligament repair associated with acute ankle fractures. Acta Orthop Belg. 2019;85(3):387–91. 31677637

[47] Slim K, et al. Methodological index for non-randomized studies (minors): development and validation of a new instrument. ANZ J Surg. 2003;73(9):712–6. 12956787 10.1046/j.1445-2197.2003.02748.x

[48] Sogbein OA, et al. Early radiographic outcomes following deltoid ligament repair in bimalleolar equivalent ankle fractures. Foot Ankle Surg. 2022;28(6):720–5. 34493449 10.1016/j.fas.2021.08.007

[49] Stromsoe K, et al. The repair of a ruptured deltoid ligament is not necessary in ankle fractures. J Bone Joint Surg Br. 1995;77(6):920–1. 7593106

[50] Sun X, et al. Does routinely repairing deltoid ligament injuries in type B ankle joint fractures influence long term outcomes? Injury. 2018;49(12):2312–7. 30526926 10.1016/j.injury.2018.11.006

[51] Tejwani NC, et al. Are outcomes of bimalleolar fractures poorer than those of lateral malleolar fractures with medial ligamentous injury? J Bone Joint Surg Am. 2007;89(7):1438–41. 17606780 10.2106/JBJS.F.01006

[52] Wang J, et al. The role of deltoid ligament repair in ankle fractures with syndesmotic instability: a systematic review. J Foot Ankle Surg. 2021;60(1):132–9. 33218869 10.1053/j.jfas.2020.02.014

[53] Wang X, et al. Treatment of medial malleolus or pure deltoid ligament injury in patients with supination-external rotation type iv ankle fractures. Orthop Surg. 2017;9(1):42–8. 28296225 10.1111/os.12318 PMC6584467

[54] Warner SJ, et al. The diagnostic accuracy of radiographs and magnetic resonance imaging in predicting deltoid ligament ruptures in ankle fractures. HSS J. 2019;15(2):115–21. 31327941 10.1007/s11420-018-09655-x PMC6609669

[55] Woo SH, Bae SY, Chung HJ. Short-term results of a ruptured deltoid ligament repair during an acute ankle fracture fixation. Foot Ankle Int. 2018;39(1):35–45. 29078057 10.1177/1071100717732383

[56] Wright JG, Swiontkowski MF, Heckman JD. Introducing levels of evidence to the journal. J Bone Joint Surg Am. 2003;85(1):1–3. 12533564

[57] Wu K, et al. Evaluation of transsyndesmotic fixation and primary deltoid ligament repair in ankle fractures with suspected combined deltoid ligament injury. J Foot Ankle Surg. 2018;57(4):694–700. 29661674 10.1053/j.jfas.2017.12.007

[58] Yu GR, et al. Repair of the acute deltoid ligament complex rupture associated with ankle fractures: a multicenter clinical study. J Foot Ankle Surg. 2015;54(2):198–202. 25618804 10.1053/j.jfas.2014.12.013

[59] Zeegers AV, van der Werken C. Rupture of the deltoid ligament in ankle fractures: should it be repaired? Injury. 1989;20(1):39–41. 2592064 10.1016/0020-1383(89)90043-0

[60] Zhao HM, et al. Surgical treatment of ankle fracture with or without deltoid ligament repair: a comparative study. BMC Musculoskelet Disord. 2017;18(1):543. 29268724 10.1186/s12891-017-1907-4 PMC5740931

