# Supplementary material for: A systematic review on how to diagnose deltoid ligament injuries—are we missing a uniform standard?
Source: BMC Musculoskelet Disord. 2024 Oct 3;25:782. doi: 10.1186/s12891-024-07869-1 (PMC11450994; doi:10.1186/s12891-024-07869-1)
Supplement: Supplementary file 1 — Supplementary Material 1. [file 12891_2024_7869_MOESM1_ESM.pdf]

## Search strategy

**PubMed (Medline)**

("Ligaments"[MESH] OR "Ligament*"[tiab]) AND ("Deltoid*" OR ("Medial"[tiab] AND ("Ankle"[tiab] OR "Spring"[tiab] OR "tender*"[tiab]))) AND ("Wounds and Injuries"[Mesh] OR "Rupture"[Mesh] OR "Injur*"[tiab] OR "rupture*"[tiab] OR "Joint Instability"[Mesh] OR "Instability"[Mesh] OR "Imag*"[tiab] OR "Diagno*"[tiab] OR "Stress, Mechanical"[Mesh] OR "Ankle Joint/diagnostic imaging"[MAJR] OR "Stress"[tiab] OR "test"[tiab])

**Embase/ Ovid**

("ligament"/ OR "ligament*"/.ti,ab.) AND ("Deltoid*" OR (“medial collateral ligament”/.ti,ab. AND ("Ankle"/.ti,ab. OR "Spring"/.ti,ab. OR "tender*"/.ti,ab.))) AND (exp Wound/ OR exp Injury/ OR exp Rupture/ OR "Injur*"/.ti,ab. OR "rupture*"/.ti,ab. OR (exp Joint/ AND exp Instability/) OR exp Instability/ OR "Imag*"/.ti,ab. OR "Diagno*"/.ti,ab. OR exp Stress/ OR exp Mechanical/ OR (exp Ankle/ AND exp Joint AND exp diagnostic/ AND exp imaging/ OR "Stress"/.ti,ab. OR "test"/.ti,ab.)

**Scoupus**

("Ligaments" OR TITLE-ABS("Ligament*")) AND ("Deltoid*" OR (TITLE-ABS("Medial") AND (TITLE-ABS("Ankle") OR TITLE-ABS("Spring") OR TITLE-ABS("tender*")))) AND (TITLE-ABS("Wounds and Injuries”) OR TITLE-ABS("Rupture") OR TITLE-ABS("Injur*") OR TITLE-ABS("rupture*") OR TITLE-ABS("Joint Instability") OR TITLE-ABS("Instability") OR TITLE-ABS("Imag*") OR TITLE-ABS("Diagno*") OR TITLE-ABS("Stress”) OR TITLE-ABS(“Mechanical") OR (TITLE-ABS("Ankle Joint”) AND TITLE-ABS(“diagnostic imaging")) OR TITLE-ABS("Stress") OR TITLE-ABS("test"))

## Central

| | | |
|---|---|---|
| #1 | MeSH descriptor: [Ligaments] explode all trees | MeSH |
| #2 | "ligament*":ti,ab,kw | Limits |
| #3 | "Deltoid*" OR ("Medial":ti,ab,kw AND ("Ankle":ti,ab,kw OR "Spring":ti,ab,kw OR "tender*":ti,ab,kw)) | Limits |
| #4 | MeSH descriptor: [Wounds and Injuries] explode all trees | MeSH |
| #5 | MeSH descriptor: [Rupture] explode all trees | MeSH |
| #6 | "Injur*":ti,ab,kw OR "rupture*":ti,ab,kw | Limits |
| #7 | MeSH descriptor: [Joint Instability] explode all trees | MeSH |
| #8 | "Instability":ti,ab,kw OR "Imag*":ti,ab,kw OR "Diagn*":ti,ab,kw | Limits |
| #9 | MeSH descriptor: [Stress, Mechanical] explode all trees | MeSH |
| #10 | MeSH descriptor: [Ankle Joint] explode all trees | MeSH |
| #11 | MeSH descriptor: [Diagnostic Imaging] explode all trees | MeSH |
| #12 | "Stress":ti,ab,kw OR "test":ti,ab,kw | Limits |
| #13 | (#1 OR #2) AND (#3) AND (#4 OR #5 OR #6 OR #7 OR #8 OR #9 OR #10 OR #11 OR #12) | Limits |
| #14 | Type a search term or use the S or MeSH buttons to compose | S MeSH Limits |

## Grey literature

Opengrey.eu, ntis.gov and the Healthcare Management Information Consortium (HMIC) were screened.
