# Supplementary material for: A systematic review on how to diagnose deltoid ligament injuries—are we missing a uniform standard?
Source: BMC Musculoskelet Disord. 2024 Oct 3;25:782. doi: 10.1186/s12891-024-07869-1 (PMC11450994; doi:10.1186/s12891-024-07869-1)
Supplement: Supplementary file 3 — Supplementary Material 3. [file 12891_2024_7869_MOESM3_ESM.pdf]

Included studies			Fracture details	Diagnostics per time point						
Author (Year)	Study Design (Level of Evidence)	Qualitiy assesement (MINORS) (total of 16/24 points for non/ comparative studies)	Fractures included	Preoperative	Before ORIF	After ORIF	After ORIF + syndesmotic stabilization	Differentiation SDL/DDL	Was the Deltoid ruptur proofed by direct visualization / arthroscopy / MRI?	Did the authors differenetiate between syndesmotic and deltoid injuries
**No treatment studies**										
Harper, Marion (1988)	Retrospective case series (LoE: IV)	5/16	SER Stage IV: n=20<br>PER Stage IV: n=18<br>Masionneuve fracture dislocations: n=3	Unstressed radiographs: MCS ≥5mm<br>*(view not further specified- a.p. or mortise)*		unstressed radiographs: a.p., lateral, mortise view		No	Yes (in 4 of 42 cases)	No
Zeegers, Aldegunde (1989)	Prospective case series (LoE: IV)	5/16	Weber B: n=12<br>Weber C: n=16<br><br>SE IV: n=12<br>PE IV: n=3<br>PE III: n=7<br>PA III: n=5<br>PA IV: n=1	Unstressed radiographs in mortise view: significant MCS widening *(MCS cut off not further specified)* **and** Clinics: swelling and tenderness				No	yes (if anatomical reduction is not sufficient)	No
Johnson, D.P. (1988)	Retrospective case series (LoE: III)	8/16	Medial malleolar fractures: n= 52<br>Lateral malleolar fractures: n=173				Radiographs *(not furthr specified)*:<br>The deltoid ligament is only exposed if accurate reduction cannot be achieved, and confirmed by operative radiographs	No	Yes (if accurate reduction cannot be achieved and confirmed by operative radiographs)	No
Bi, Chun (2019)<br>*comparison of gravitiy stress test and tap test*	Prospective case series (LoE: IV)	10/16	Distal fibular fractures: n=92<br>- Type B : n= 32<br>- Type C: n= 59<br><br>SER: n= 42<br>PER: n=42<br>PAR: n=7		Gravitiy stress test: MCS>4mm **and** MCS>2mm greater than the superior joint space	Tap test: The before and after talus shift and widening of the medial clear space were compared. A positive finding was defined as an increase in the medial clear space of >2mm		No	Yes (for differentiation between SDL and DDL)	No
Tejwani, Nirmal C. (2006)	Prospective case series (LoE: IV)	9/16	SER IV fractures: n= 266<br>Bimalleolar fractures: n= 163<br>Lateral malleolar fractures: n= 103	External rotational stress test: MCS widening<br>*(MCS cut off not further specified)*				No	No	Yes
**Comparative studies (No repair/ syndesmotic repair/ DL repair)**										
Woo, Seung Hun (2017)	Retrospective cohort study (LoE: III)	16/24	Lateral malleolar fractures: n= 78<br>SER: n=62<br>PER: n=16	Clinics: pain and swelling, tenderness, echhymycosis around the deltoid ligament/ medial gutter			Gravity stress test in mortise view: MCS >4mm **or** MCS >1mm than the superior joint space<br>**or** External rotational stress test: MCS >4mm **or** MCS >1mm than the superior joint space	yes (for therapeutic purposes)	Yes (MRI was obtained for 25 of the patients, in which 22 were found to have a complete deltoid rupture; direct visualization in the repair group)	Yes
Chen, Hongfeng (2020)	Retrospective cohort study (LoE: III)	14/24	SER IV: n=63	MCS≥5mm *(radiographic assessement not further specified)*				yes (for therapeutic purposes)	No	Yes
Choi, SeongJu (2020)	Retrospective cohort study (LoE: III)	15/24	SER IV: n=34	Unstressed radiographs in mortise view:<br>MCS>6 mm<br>CT-scan: Widening of the syndesmosis				No	Yes (Only in the repair group)	Yes
Sogbein, Olawale (2021)	Retrospective cohort study (LoE: III)	14/24	Lateral malleolar fractures with associated deep deltoid ligament injuries (bimalleolar equivalent ankle fractures): n=147<br><br>Weber B: n=93<br>Weber C: n=54	Unstressed radiographs in mortise view: MCS≥5mm: The deep deltoid ligament was assumed to be ruptured		External rotational stress test: MCS widening *(MCS cut off not further specified, but patients must have had preop MCS≥5mm)*: Either nor repair, or DL repair, or syndesmotic repair, per the surgeons choice	If syndesmotic screws were placed and residual medial widening or significant talar tilt was observed, DL repair was subsequently performed	No	Yes (Only in the repair group)	Yes
Asadi, Kamran (2021)	Retrospective cohort study (LoE: III)	13/24	44-A: n=12<br>44-B: n=35<br>44-C: n=18	External rotational stress test: MCS >3mm clinical and paraclinical findings *(not further specified)*				yes (for therapeutic purposes)	Yes (Only in the repair group)	No
Stromsoe, Knut (1995)	Randomized controlled Study (LoE: I)	14/24	Distal fibular fractures: n=50<br>- Weber B: n=30<br>- Weber C: n=20	Unstressed radiographs in a.p. view: difference in width between the medial and the lateral clear space and the talar margins >3mm				No	Yes (in non suture group, when medial widening after ORIF was persistent)	No
Gu, Guanxue (2017)	Randomized controlled Study (LoE: I)	15/24	not stated (´ankle fractures´)	Stressed radiographs *(not further specified which view)* : MCS >5mm **and** confirmed rupture of deep and superficial deltoid ligaments by MRI				No	Yes (Only in the repair group)	No
Li, Ting (2020)	Retrospective cohort study (LoE: III)	8/24	Distal fibular fractures: n=71<br>- Weber B: n=42<br>- Weber C: n= 29			Stressed radiographs: MCS widening without syndesmotic widening *(Cut off MCS and radiographic imaging not further specified)*: SDL repair without fixation of the inferior tibiofibular syndesmosis	Stressed radiographs: MCS widening **and** syndesmotic widening *(Cut off MCS and radiographic imaging not further specified)*: syndesmotic fixation with screws before another stress test to confirm that a DL repair is needed	yes (for therapeutic purposes)	Yes (Only in the repair group)	Yes
Rosa, Isabel (2020)	Randomized controlled Study (LoE: I)	15/24	isolated lateral malleolar fractures: n=65	Gravitiy stress radiographs: MCS> 4mm		Positive manual stress test *(cut off values not further specified, but preopertaive MCS>4mm)*		yes (for therapeutic purposes)	No	Yes
Dagtas, Mirza Zafer (2021)	Retrospective cohort study (LoE: III)	12/24	Distal fibular fractures: n=78<br>44-B: n=38<br>44-C: n= 40	Unstressed radiographs in mortise view: MCS>4mm				No	Yes (Only in the repair group)	Yes
Zhao, Hong-Mou (2017)	Retrospective cohort study (LoE: III)	15/24	AO-type B (SER): n=49<br>AO-type C (PER/PA): n=25	Unstressed radiographs in a.p. view: MCS≥ 6mm				yes (for therapeutic purposes)	Yes (Only in the repair group)	Yes
Wang, Xu (2017)	Prospective cohort study (LoE: II)	12/24	SER type IV: 35		External rotational stress test:<br>MCS>4mm **or** MCS>1mm greater than superior joint space			No	Yes (Only in the repair group)	No
Sun, Xu (2018)	Randomized controlled Study (LoE: I)	13/24	Isolated malleolar fractures Type B: n=41		Valgus and external rotational stress test:<br>MCS widening more than two times to the space of the tibiotalar joint of the uninjured side			yes (for therapeutic purposes)	Yes	Yes
**All repaired studies**										
Hsu, Andrew (2015)	Retrospective case series (LoE: IV)	7/16	Not stated (´high-energy ankle fractures´): n=14	Stressed radiographs *(not further specified)*:<br>MCS widening and syndesmotic disruption<br>(The authors were contacted and stated that they had a very low threshold to openly explore the SDL)	External rotational stress test under Flouroscopy: Talat tilt			Yes	Yes	Yes
				Clinics:<br>- Hematoma, swelling, tenderness, bruising<br>- Dimple sign<br>- Anterior drawer test	Arthroscopic evaluation of Syndesmosis and DL (static and dynamic)<br><br>(The authors were contacted and stated that all young healthy patients and athletes who have ankle fractures were scoped)			Yes	Yes	No
Yu, Guang-rong (2015)	Prospective case series (LoE: IV)	10/16	SER Stage IV: n=42<br>PER Stage III: n=36<br>PER Stage IV: n=48<br>PA Stage III: n=5<br><br>43-B2: n=3,<br>44-B2: n=9<br>44-B3: n=48<br>44-C1: n=21<br>44-C2: n=24<br>44-C3: n=26	Unstressed radiographs in mortise view:<br>MCS≥4mm, talar subluxation<br>(diagnosis of DL rupture: MCS>4mm<br>Indication for DL repair: MCS>5mm<br>indicative for DL rupture: talar subluxation)<br>Indicative clinics: swelling, ecchymosis, tenderness<br><br>Conducted diagnostics: radiographs, CT-scan, MRI (96 patients; 85% had a complete deltoid rupture)		Operative repair was **also** indicated, if persistend medial ankle instability was evident on external rotational stress testing after fracture fixation.<br>A medial clear space >1mm after fracture fixation was considered indicative of entrapment of the medial soft tissue structures. Medial exploration with repair of the deltoid ligament was indicated		No	Yes	No
Park, Young Hwan (2020)	Retrospective cohort study (LoE: III)	8/16	SER IV: n=106<br>PA III: n=4<br>PER III: n=5<br>PER IV: n=16				Manual dorsiflexion and external rotational stress test were completed with intraoperative fluoroscopy at the end of the surgical procedure. A MCS≥5mm displays g a complete DL rupture, indicating DL epair. (The MCS was measured manually on the fluoroscopy monitor using a ruler, and the magnification effects were normalized on the basis of screw diameter used to fix malleolar fracture or syndesmosis)	No	Yes	Yes
Mansur, Nacime (2021)	Retrospective cohort study (LoE: III)	8/16	Acute (less than two weeks) ankle fractures and a medial ligament injury suspicion. Acute distal syndesmotic lesions with associated deltoid rupture were also included: n=20	Radiographs (stressed or unstressed): MCS>4mm<br>Clinics: pain at the medial malleolus	Arthroscopy was performed in all ankle fracture cases*<br>Arthroscopic evaluation of the dletoid: (1) ´Open book´ appearance at anterior border of the tibial malle- olus; (2) ability to insert a 4mm instrument in the tibial medial corner or in the medial clear space (between the talus and the me- dial malleolus) and (3) direct visualization of ruptured superficial or deep deltoid fibers<br><br>*author was contacted			Yes (for therapeutic purposes)	Yes	Yes
Diab, Hossam (2017)	Prospective case series (LoE: IV)	8/16	Pronation ankle injuries, lateral malleolar fractures, and deltoid ligament rupture: n=27		Stress screening/ stress radiographs under image intensification: MCS>4mm		Exclusion criteria: syndesmotic disruption requiring fixation	No	yes	Yes
Shen, Jian-Jian (2019)	Prospective case series (LoE: IV)	7/16	44-B.2.1: n=9<br>44-B.3.1: n=16<br>44-C.1.1: n=7<br>44-C.2.1: n=2<br><br>SER type IV: n=11<br>PER tpye III: n=9<br>PER type IV: n=12<br>PA type IV: n=2				External rotational stress test: MCS>5mm	No	yes	Yes
**Other**										
Jones, Christopher (2015)	Retrospective cohort study (LoE: III)	13/24	Isolated SER Stage IV, bimalleolar equivalent fractures: n=27	Gravity stress test: MCS >5mm **or**<br>External rotational stress test: MCS>5mm				No	Yes (only in the reapair group)	No
Li, Bohua (2019)	Retrospective cohort study (LoE: III)	14/24	44-B3.1 (SER IV): n=23				External rotation stress test: DL repair if increased lateral talar tilt **or** MCS>5mm persist	No	Yes (only in the reapair group)	No
Lee, Tae Hoon (2016)	Prospective cohort study (LoE: II)	11/24	Isolated lateral malleolar fractures: n=35	Unstressed radiographs in a.p., mortise, lateral view *(cut off criteria not further specified)* MRI: Classification of anterior and posterior DL damage: Grade 0= no damage; Grade I= periligamentous edema present; Grade II= a partial tear presenting as laxity, irregular contour, or partial discontinuity with hyperintensity of the ligament<br>Grade III= a complete ligament tear	Valgus Stress Test:<br>- displacement≥2mm<br>- shortening<br>- rotation<br>- Talar tilt≥2°<br>- MCS≥5mm<br><br>≥1 result sets the indication for ORIF	Valgus Stress Test:<br>- Talar tilt of ≥2°<br>- MCS ≥5mm<br>(Repetition of valgus stress test under fluoroscope: Patients with residual valgus instability were classified as the high-grade unstable group	´Ecxclusion of obvious syndesmotic instability´	yes (MRI)	Yes (MRI + exposed in the repair group)	Yes
Baird, Robert (1987)	Retrospective cohort study (LoE: III)	10/24	SER IV: n=13<br>PER III: n=6<br>PER IV: n=5	Unstressed radiographs in mortise view: MCS wider than superior joint space **or** MCS>4mm in case of talus subluxation or dislocation from beneath the tibia Clinics: swelling, tenderness in the region of the deltoid ligament			Unstressed radiographs in mortise view: MCS widening *(cut off MCS not further specified)*	No	Yes (only in the reapair group)	Yes
De Souza, L.J. (1985)	Retrospective case series (LoE: IV)	12/24	Weber B: n=12<br>Weber C: n= 10			Radiographs: MCS widening<br>*(radiographic view and cut off MCS not further specified)*		No	Yes (only in the reapair group)	No
Wu, Kai (2017)	Randomized controlled Study (LoE: I)	16/24	SER: n=22<br>PER: n=18<br>PAR: n=11<br><br>Weber B: n=19<br>Wber C: n=32	Tap test: MCS >4mm **and** talus outward shifting (Indication for inclusion in study part ´tap test´)		Direct visualization<br>(Indication for inclusion in study part ´outcome´)		No	Yes	Yes
Chen, Pei-Yu (2008)	Prospective cohort study (LoE: II)	7/16	Isolated lateral malleolar fractures at the syndesmotic level: n=15	Unstressed radiographs in a.p. view: MCS widening<br>*(cut off MCS not further specified)*<br>Sonographic evaluation of the deltoid ligaments<br>Clinics: medial ankle tenderness, ecchymosis<br><br>(Surgery was performed if sonography showed complete rupture of the deep deltoid ligament)				yes (sonography)	Yes (only those with a sonographical total rupture were treated)	No
